# Supplementary material for: Hsp90 provides a platform for kinase dephosphorylation by PP5
Source: Nat Commun. 2023 Apr 17;14:2197. doi: 10.1038/s41467-023-37659-7 (PMC10110553; doi:10.1038/s41467-023-37659-7)
Supplement: Supplementary file 1 — Supplementary information [file 41467_2023_37659_MOESM1_ESM.pdf]

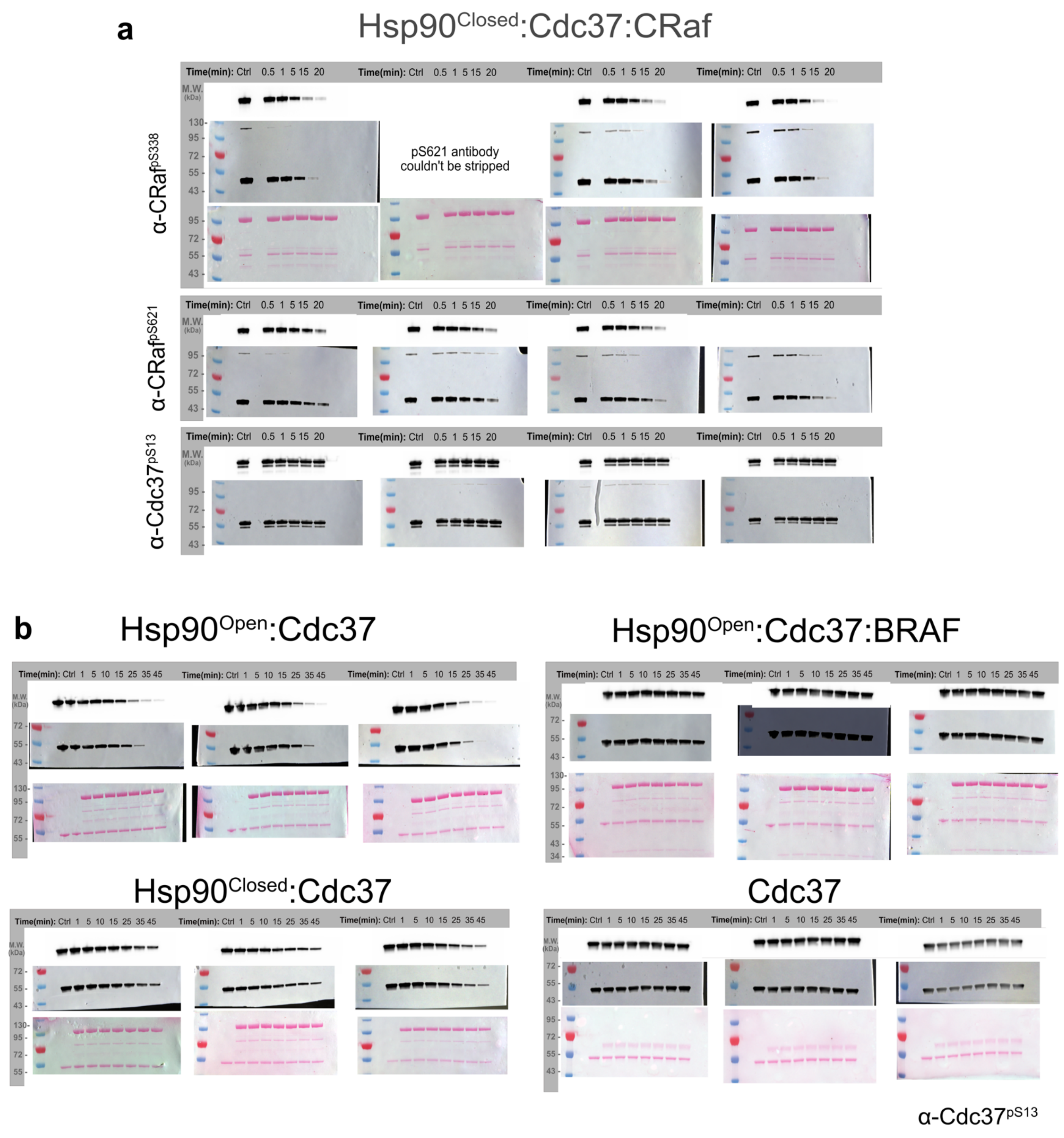

Supplementary Fig. 1. Figure 1 Western blot replicate data. a Purified CRaf-complex (1.5 μM) was incubated with PP5 (75 nM) at 25°C, ran on a gel transferred to a nitrocellulose membrane, ponzo stained and blotted for α-CRaf^{pS338}, α-CRaf^{pS621}, and α-Cdc37^{pS13}. **b** Reconstituted complex (3 μM) was incubated with PP5 (750 nM) at 25°C, ran on a gel, transferred to a nitrocellulose membrane, ponzo stained and blotted for α-Cdc37^{pS13}. Equal amounts of protein can be seen in ponzo stained membrane throughout experimental timepoints. Blot strips were processed in parallel, and all were normalized by the phosphorylation signal of the internal control which has not been in contact with PP5.

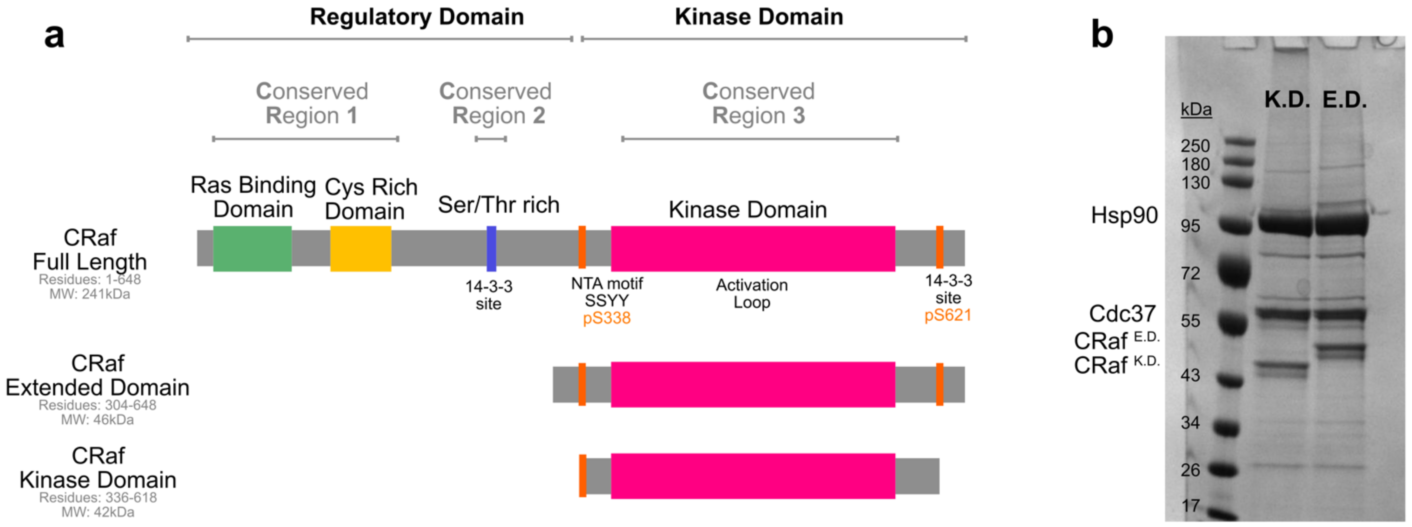

Supplementary Fig. 2: CRaf topology and constructs. a CRaf consists of a Regulatory and Kinase domain. The CRaf regulatory domain contains a Ras Binding Domain, a Cysteine Rich Domain, and important phosphorylation sites such as pS259 and pS621 which bind 14-3-3 chaperones. The CRaf kinase domain consists of two lobes, the beta sheet heavy N-lobe and alpha helical C-lobe. ATP binds between these two lobes. Important phosphorylation sites around the kinase domain are highlighted in orange and consist of the N-terminal pS338 and the C-terminal pS621. Two shorter CRaf constructs were used to increase solubility of CRaf and enable further biochemistry. The Extended domain ($CRaf^{ED}$) was used for all biochemical experiments, while the Kinase domain ($CRaf^{KD}$) was used for structural characterization. **b** Hsp90:Cdc37:CRaf complexes were purified from Expi HEK293 cells, a process repeated with similar results more than five times.

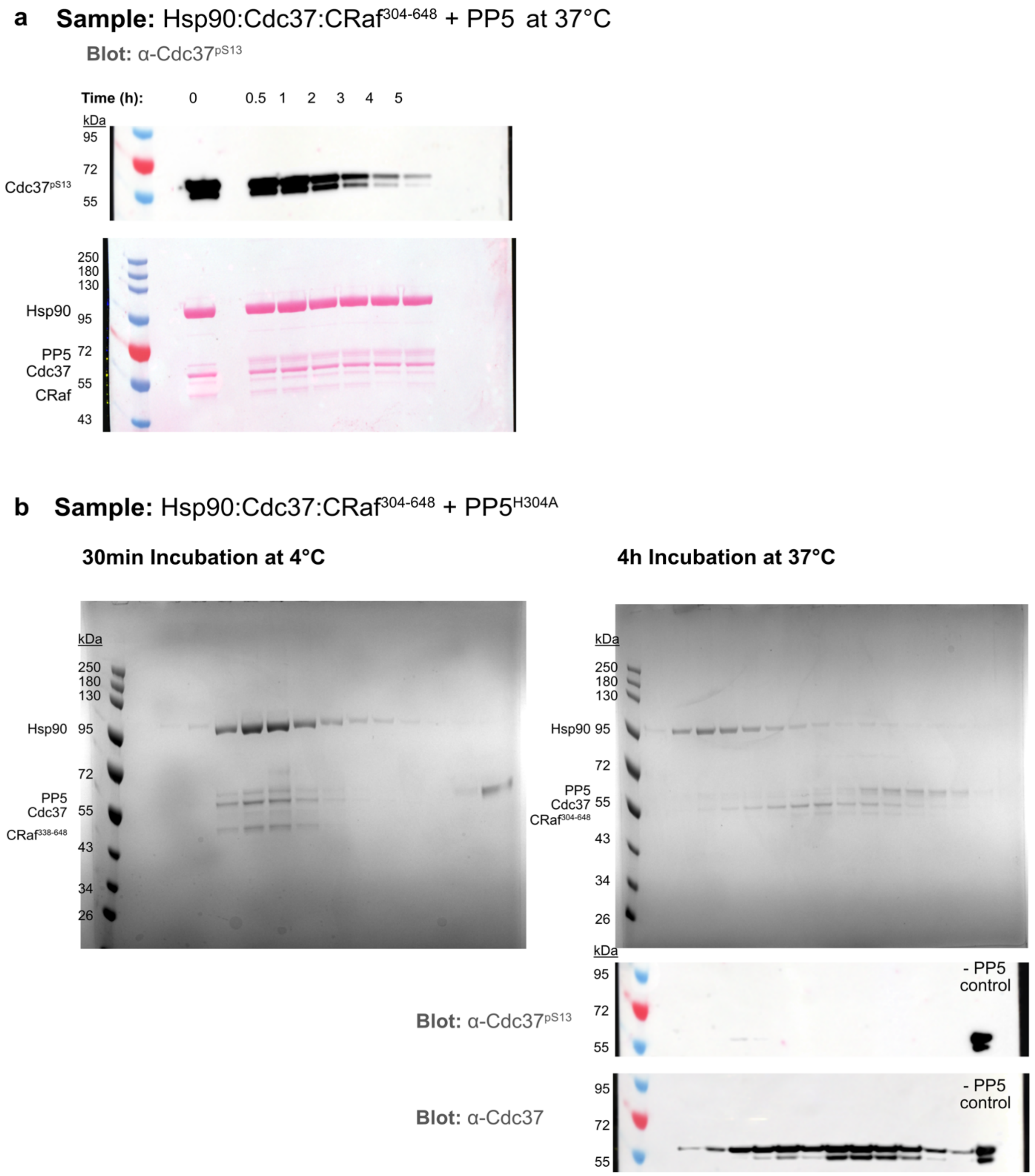

Supplementary Fig. 3: Cdc37 becomes dephosphorylated by PP5 at high temperatures.
a Hsp90:Cdc37pS13:CRaf$^{304-648}$ complex (2 μM) was incubated with PP5 (2 μM) at 37°C. The reaction was quenched by SDS:DTT at differing timepoints. The sample was then run on a gel, transferred to a nitrocellulose membrane, ponzo stained and blotted for α-Cdc37^{pS13}. These results were seen three times. **b** Hsp90:Cdc37:CRaf:PP5 complexes were run through a Superdex 200 column after incubation for 30 min at 4°C or for 4h at 37°C. Complex dissociation and PP5 dephoshorylation can be seen in the gel trace of the 37°C incubated sample, which show larger complexes on the left side of the gel and smaller complexes on the right side of the gel. Slight Cdc37^{pS13} phosphorylation can be seen where the complex remains intact as seen by the Western blots provided. This experiment was repeated twice.

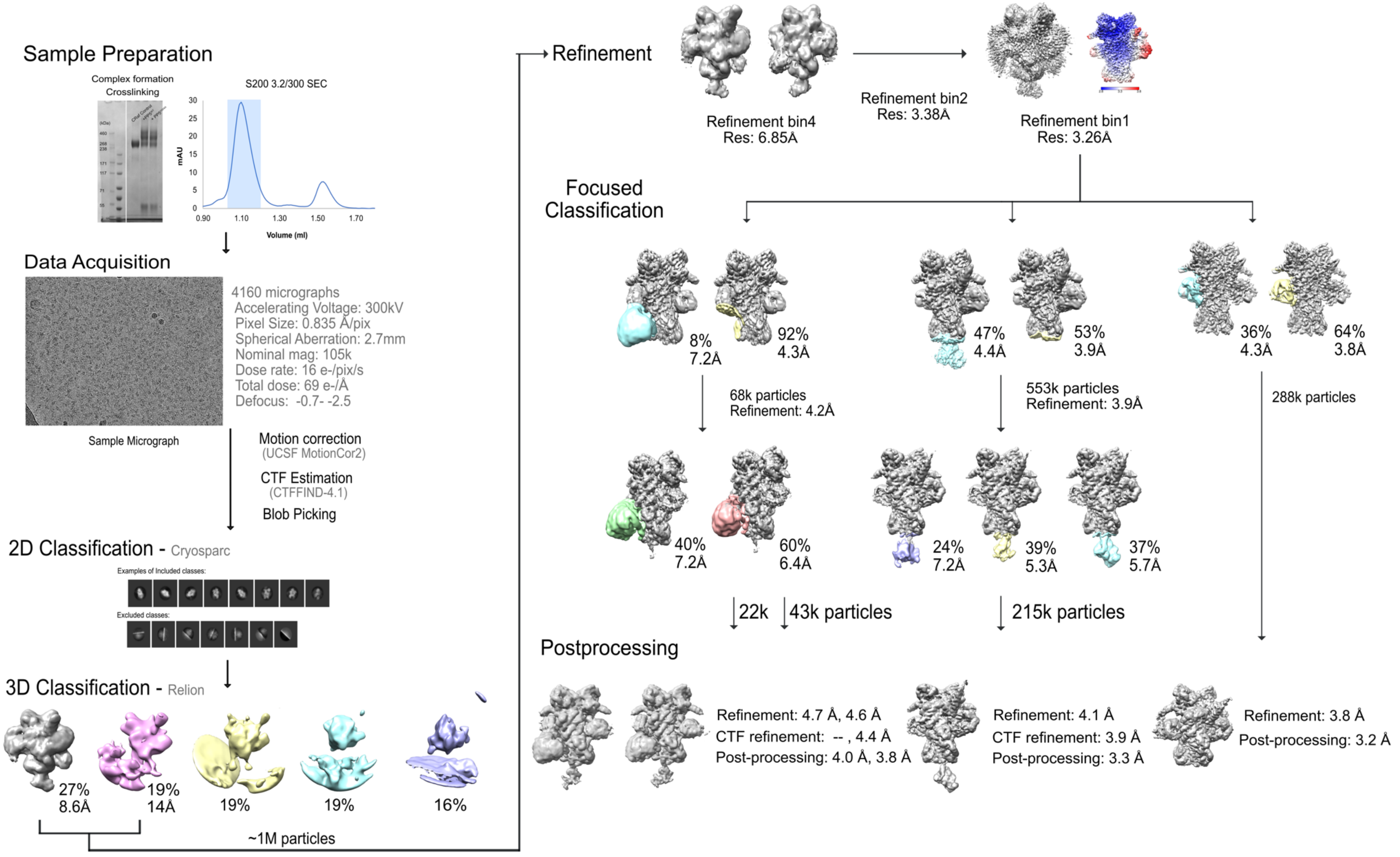

Supplementary Fig. 4: Sample preparation and cryo-EM data processing. The human Hsp90:Cdc37:CRaf complex was purified from yeast, and human PP5^{H304A} was purified from E. coli. The Hsp90:Cdc37:CRaf complex (2 μM) was mixed with PP5^{H304A} (6 μM) for 30 min on ice, and then brought to room temperature and crosslinked with glutaraldehyde (0.05%, 15 min, quenched with 50 mM Tris pH 7.8). The crosslinked complex was then run over an S200 column (3.2/300) to remove excess PP5. The fractions from the major peak (~1.1 mL) were collected and concentrated 3 fold. This sample was frozen on PEG Amino functionalized gold carbon quantifoil grids using a Vitrobot (10C, 100% humidity, 30s Wait Time, 3s Blot Time, -2 Blot Force). 4160 micrographs were acquired using a Krios microscope (105,000X magnification). The images were then motion corrected (MotionCor2), and their CTF estimated (CTFFIND-4.1). Micrographs with a CTF fit <5Å were kept for cryoSPARC gaussian blob picking. The particles were then 2D classified to remove high resolution artifact particles and ensure sample quality. Selected 2D classes were then imported into RELION (using csparc2star by D. Asarnov) for further 3D classification. One round of classification led to ~1M Hsp90-like particles, which were then refined and unbinned. Focused classification with subtraction, Refinement and Postprocessing led to the final maps used for composite map creation and model fitting.

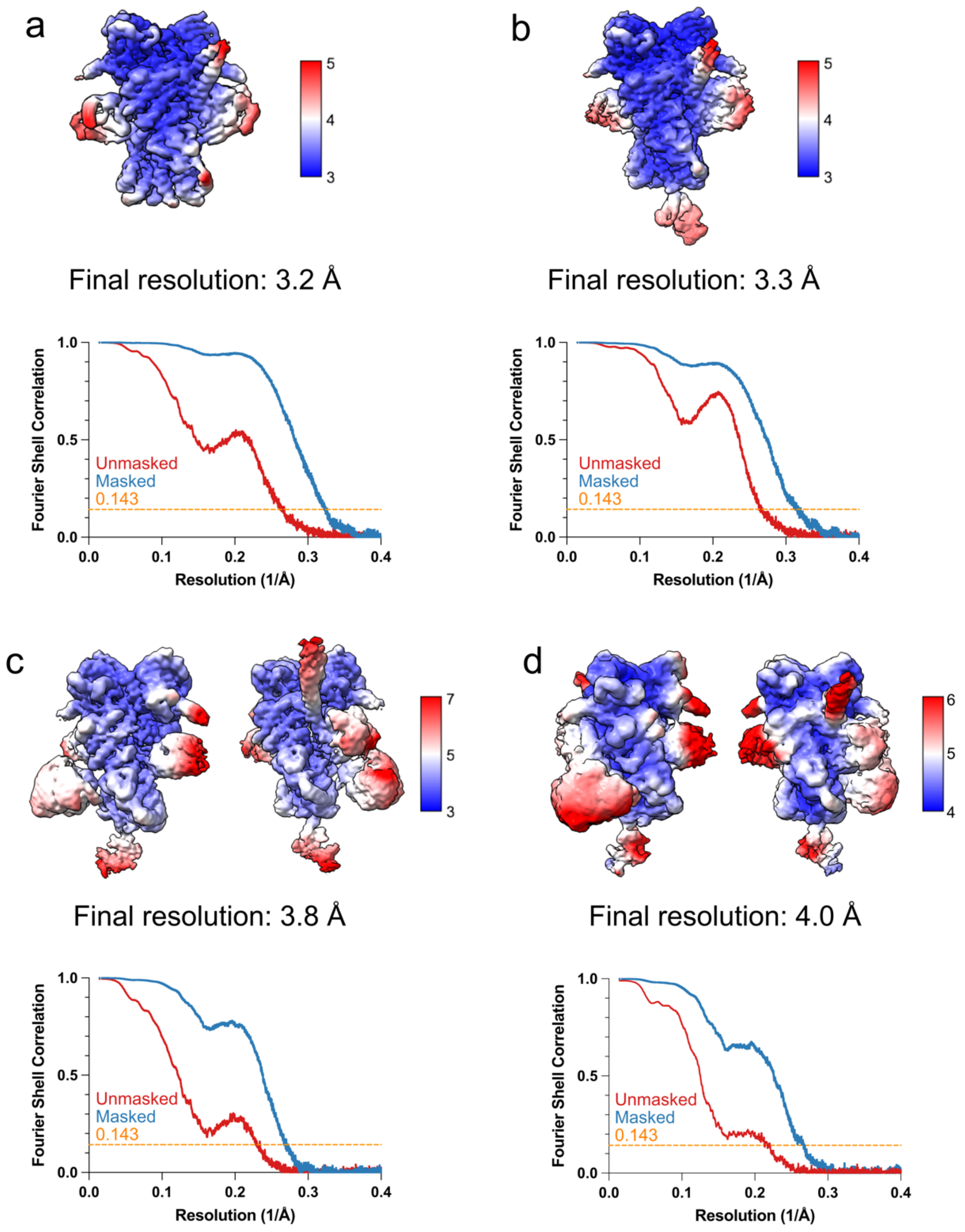

Supplementary Fig. 5: Focused classification on the CRaf N-lobe (**a**), the TPR domain of PP5 (**b**), and two alternate PP5 conformations (**c,d**) yielded the final maps used to create the two composite models in this work. The maps included **a, b** and either **c** or **d**. The volumes shown here were post-processed in RELION to give the final resolutions reported. The FSC curves were obtained using the phenix software (Mtriage).

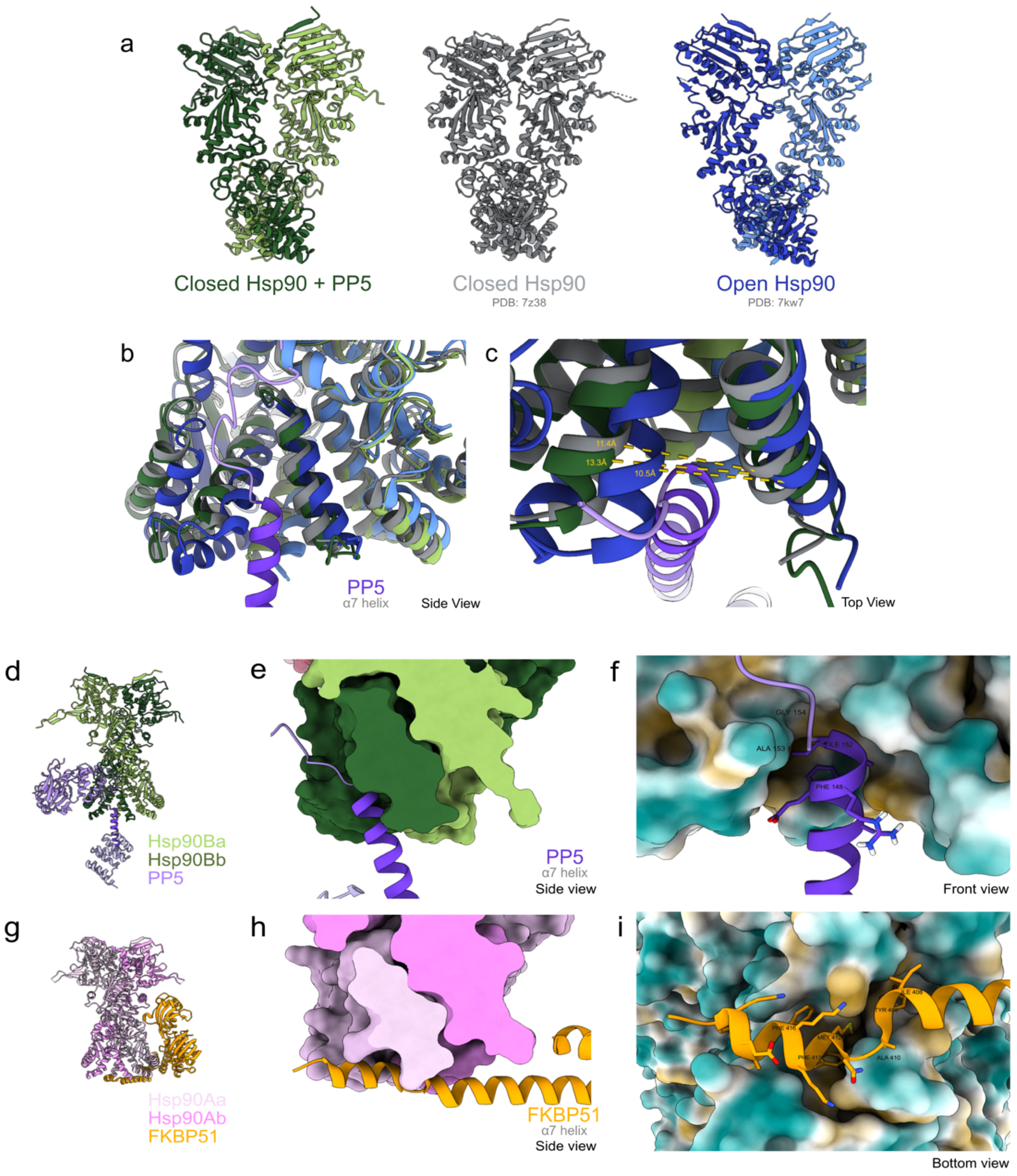

Supplementary Fig. 6: Comparison between Hsp90 conformations, and differently binding cochaperones. **a** Closed Hsp90 dimer as seen in our current work is contrasted with the Hsp90:Cdc37:CRaf complex (PDB: 7z38 [PDB DOI: 10.2210/pdb7Z38/pdb]) and the loading complex semi-open Hsp90 dimer (PDB: 7kw7 [PDB DOI: 10.2210/pdb7KW7/pdb]). **b,c** A closer view of the Hsp90^{CTD} shows the rearrangements that occur between the Hsp90 CTD groove that binds PP5s α7 helix. The Hsp90 CTD groove varies in size (Hsp90^{closed}:PP5 > Hsp90^{closed} > Hsp90$^{semi\text{-}open}$); Hsp90^{closed}:PP5 allows PP5 binding in its enlarged CTD groove. **d,g** Hsp90 interacts with PP5 and FKBP51 via TPR domain:Hsp90 C-terminal interactions. **e,h** The cochaperone's α7 helices interact with the Hsp90 C-terminal groove at an almost ~90° angle difference from each other. **f,i** Numerous FKBP51 α7 helix hydrophobic residues are buried in the Hsp90:FKBP51 interface, while only two hydrophobic residues are buried in the Hsp90:PP5 interface.

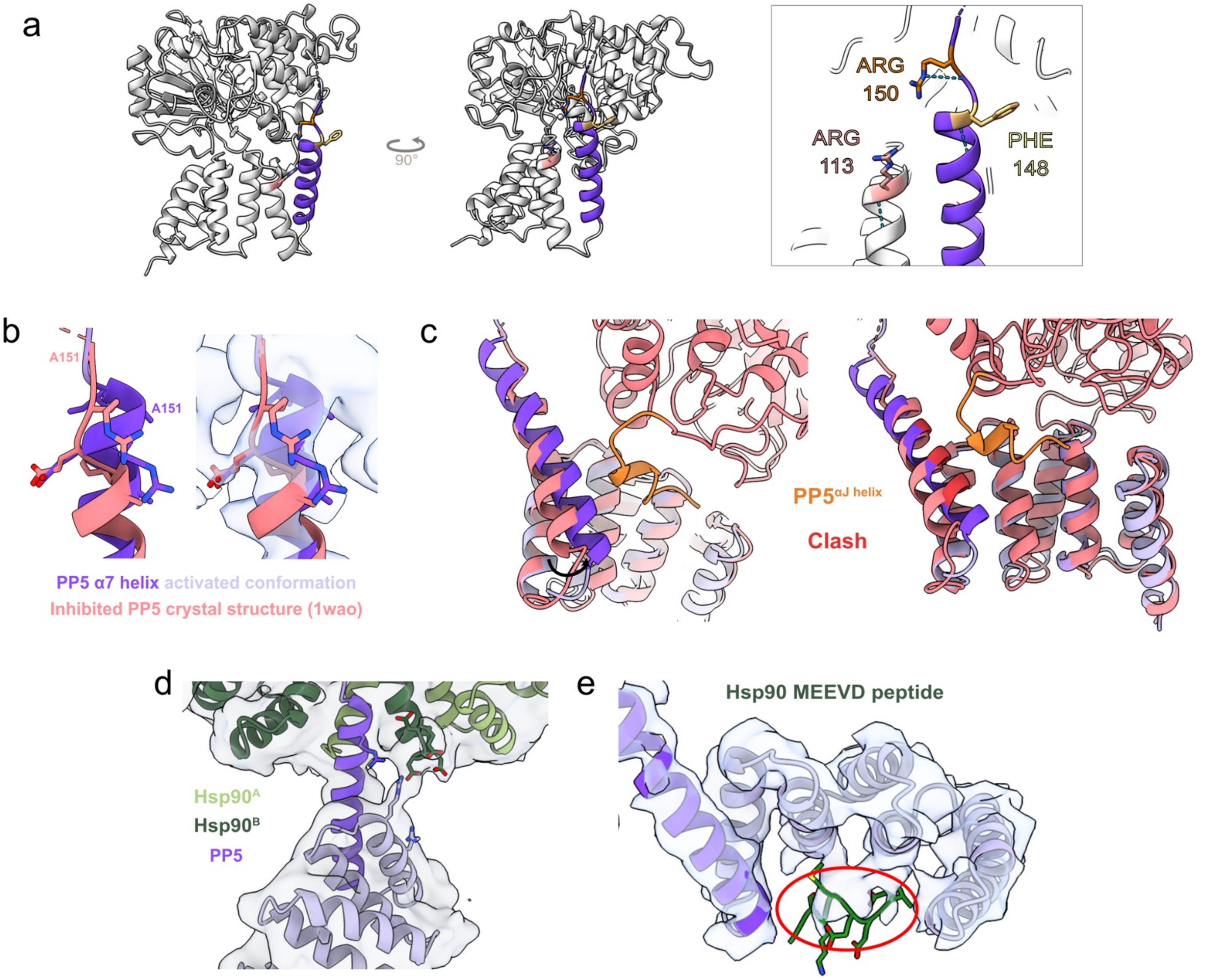

Supplementary Fig. 7: Hsp90 binding leads to PP5 TPR domain rearrangement. a The PP5 TPR mutants are more than 8Å away from the PP5 catalytic site. The $\alpha 7$ helix on the TPR domain binds to Hsp90's C-terminal domains. **b** PP5 $\alpha 7$ helix binding to $Hsp90^{CTD}$ leads to the elongation of the PP5 $\alpha 7$ helix as it makes stabilizing interactions with Hsp90. **c** Overlay of PP5's TPR domain in an inhibited (PDB: 1wao) or active state shows PP5 $\alpha 7$ helix movement towards the PP5 αJ helix of PP5's catalytic domain. Potential clash (red) between these two helices might contribute to αJ helix inhibition release. **d** Density can be seen for the interaction between the negatively charged Hsp90 tail and the positively charged patch on TPR domain. **e** Partial density can be seen for the Hsp90 MEEVD peptide as it interacts with PP5's basic patch. A homologous structure of a TPR-MEEVD interaction was docked to highlight the location of key MEEVD binding residues on PP5. (PDB: 6q3q [PDB DOI: 10.2210/pdb6Q3Q/pdb]).

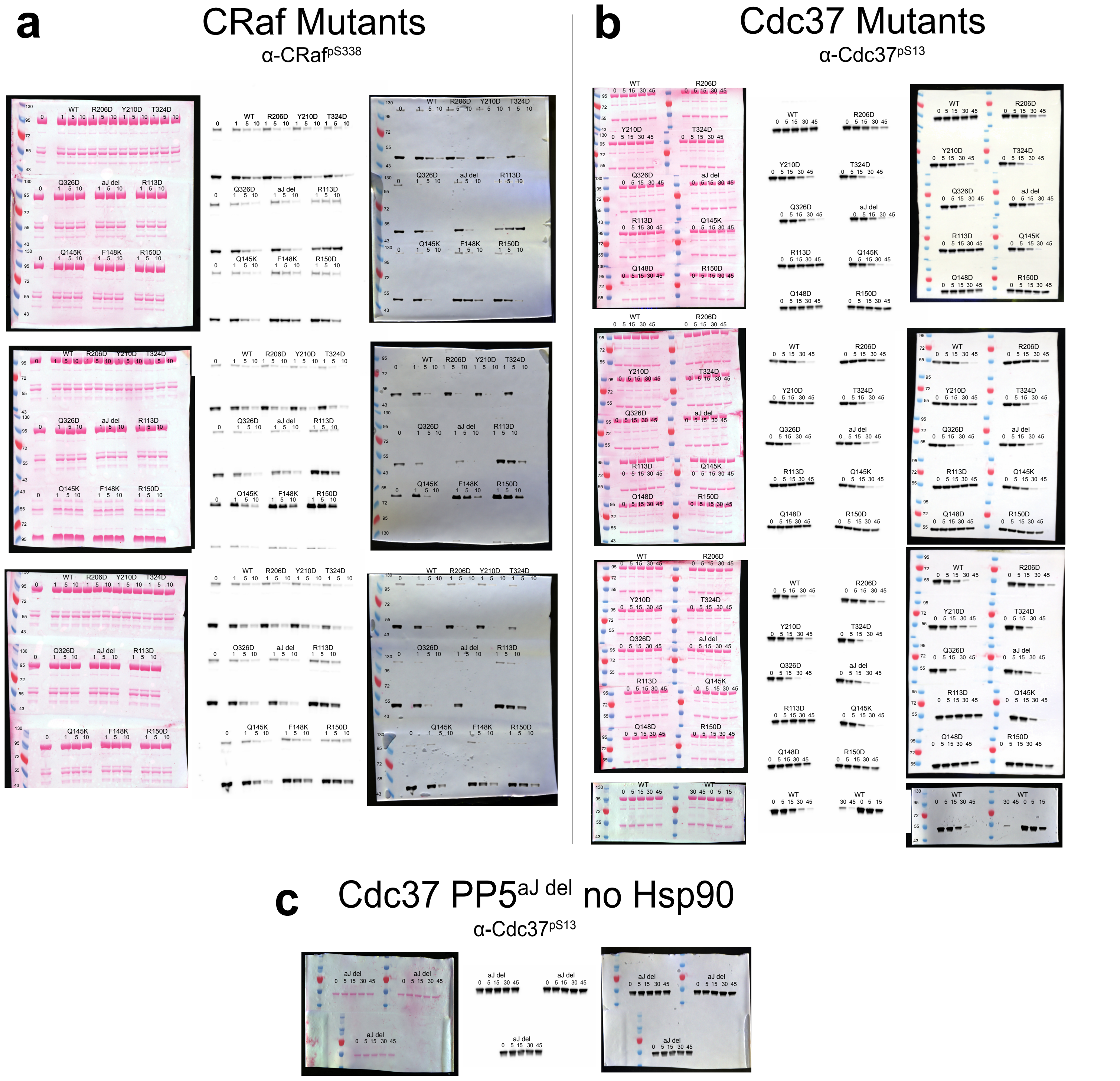

Supplementary Fig. 8: Mutant western blot replicate data. PP5 mutants were tested for rates of dephosphorylation as described in Supplementary Fig. 1. **a** Mammalian purified $Hsp90^{closed}$:Cdc37:$CRaf^{ED}$ (3 μM) was incubated with PP5 mutant (150 nM). **b** E. coli purified, CK2 phosphorylated $Cdc37^{pS13}$ (3 μM) was incubated with Hsp90 and PP5 mutants (750 nM). Additional mutants not considered in the manuscript are included here. **c** Cdc37 (3 μM) was incubated with $PP5^{\alpha J\ del}$ (750 nM) in the absence of Hsp90.

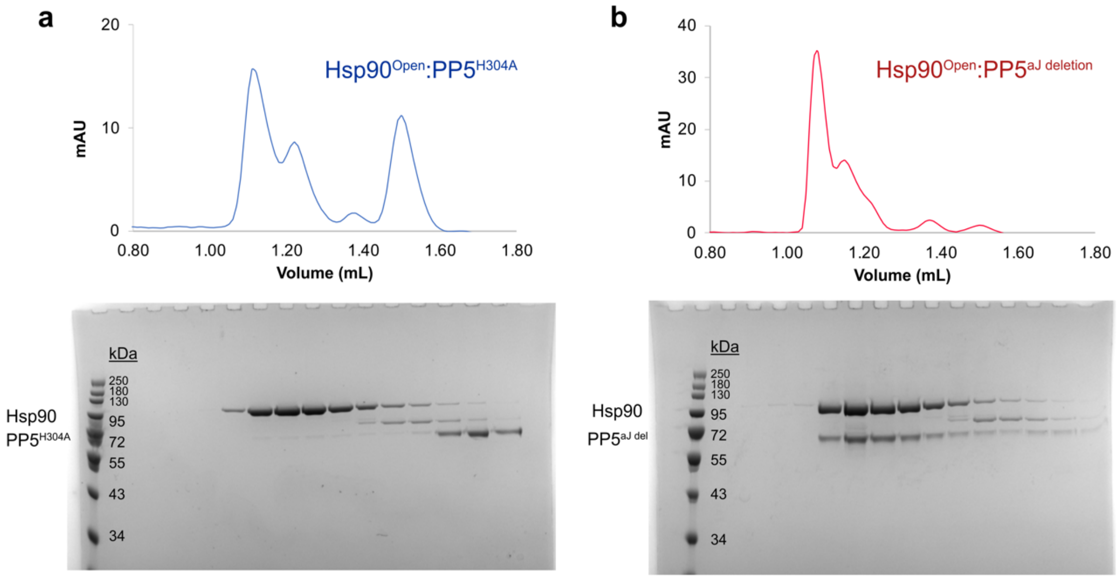

Supplementary Fig. 9: αJ helix Truncation leads to increased Hsp90:PP5 complex coelution. Hsp90 dimer (6 μM) was incubated with either catalytically dead $PP5^{H304A}$ or $PP5^{\alpha J\ del}$ (6 μM) at 4°C for 30 min before loading the sample onto the S200 3.2/300 column in SEC buffer (20 mM HEPES, 50 mM KCl, 10m $MgCl_{2,}$ 1 mM EDTA, 1 mM TCEP) and allowed to flow through at a flow rate of 0.04ml/min. 50 μL aliquots were eluted and ran on SDS gels for component visualization. **a** $PP5^{H304A}$ loosely coelutes with Hsp90, while **b** $PP5^{\alpha J\ del}$ strongly coelutes with Hsp90. This result was repeated twice.
