# Supplementary material for: Hsp90 provides a platform for kinase dephosphorylation by PP5
Source: Nat Commun. 2023 Apr 17;14:2197. doi: 10.1038/s41467-023-37659-7 (PMC10110553; doi:10.1038/s41467-023-37659-7)
Supplement: Supplementary file 3 — Additional Supplementary Files [file 41467_2023_37659_MOESM3_ESM.pdf]

File name: Supplementary Movie 1

Description: Movie shows a morph between inhibited and activated PP5 conformations. The PP5 TPR domain binds the Hsp90 CTD groove and allows the PP5 catalytic domain to reach towards the Hsp90 bound CRaf Clobe.

File name: Supplementary Movie 2

Description: 3D variability analysis of the Hsp90:Cdc37:CRaf:PP5 complex shows heterogeneous PP5 binding conformations.

File name: Supplementary Movie 3

Description: 3D variability analysis allows for the visualization of density extending from the C-terminal CRaf alpha helix towards the PP5 active site.
