# Supplementary material for: Hsp90 provides a platform for kinase dephosphorylation by PP5
Source: Nat Commun. 2023 Apr 17;14:2197. doi: 10.1038/s41467-023-37659-7 (PMC10110553; doi:10.1038/s41467-023-37659-7)
Supplement: Supplementary file 7 — Reporting Summary [file 41467_2023_37659_MOESM7_ESM.pdf]

nature portfolio

Corresponding author(s): David A. Agard

Last updated by author(s): Mar 12, 2023

Reporting Summary

Nature Portfolio wishes to improve the reproducibility of the work that we publish. This form provides structure for consistency and transparency in reporting. For further information on Nature Portfolio policies, see our Editorial Policies and the Editorial Policy Checklist.

Statistics

For all statistical analyses, confirm that the following items are present in the figure legend, table legend, main text, or Methods section.

n/a	Confirmed	
☐	☒	The exact sample size (*n*) for each experimental group/condition, given as a discrete number and unit of measurement
☐	☒	A statement on whether measurements were taken from distinct samples or whether the same sample was measured repeatedly
☐	☒	The statistical test(s) used AND whether they are one- or two-sided<br>*Only common tests should be described solely by name; describe more complex techniques in the Methods section.*
☒	☐	A description of all covariates tested
☐	☒	A description of any assumptions or corrections, such as tests of normality and adjustment for multiple comparisons
☐	☒	A full description of the statistical parameters including central tendency (e.g. means) or other basic estimates (e.g. regression coefficient) AND variation (e.g. standard deviation) or associated estimates of uncertainty (e.g. confidence intervals)
☐	☒	For null hypothesis testing, the test statistic (e.g. F, t, r) with confidence intervals, effect sizes, degrees of freedom and P value noted<br>*Give P values as exact values whenever suitable.*
☒	☐	For Bayesian analysis, information on the choice of priors and Markov chain Monte Carlo settings
☒	☐	For hierarchical and complex designs, identification of the appropriate level for tests and full reporting of outcomes
☒	☐	Estimates of effect sizes (e.g. Cohen's *d*, Pearson's *r*), indicating how they were calculated

Our web collection on statistics for biologists contains articles on many of the points above.

Software and code

Policy information about availability of computer code

Data collection	Cryo-EM data collected on a Titan Krios (Thermo Fischer Scientific) electron microscope (300kV). A K3 direct electron detector (Gatan) equipped with a Bioquantum energy filter (Gatan) set to a slit width of 20eV. Serial EM v.3.8-beta was used for automatic data collection.
Data analysis	EM data processing was done using CryoSparc v3.3.2 , RELION v.3.1.3, UCSF MotionCor2, CTFFIND v.4.1, UCSF Chimera v. 1.15, and UCSF ChimeraX v.1.2.5. Model building was done using Rosetta v.3.11, ISOLDE 1.0b3, and Phenix 1.20.1-4487.<br><br>Western blot was quantified using ImageJ v.1.53. Graphical data was plotted and statistical analysis was performed using Prism (GraphPad) 9.3.1 (350).

For manuscripts utilizing custom algorithms or software that are central to the research but not yet described in published literature, software must be made available to editors and reviewers. We strongly encourage code deposition in a community repository (e.g. GitHub). See the Nature Portfolio guidelines for submitting code & software for further information.

Data

Policy information about availability of data

All manuscripts must include a data availability statement. This statement should provide the following information, where applicable:

- Accession codes, unique identifiers, or web links for publicly available datasets
- A description of any restrictions on data availability
- For clinical datasets or third party data, please ensure that the statement adheres to our policy

The cryo-EM maps generated in this study were deposited in the Electron Microscopy DataBank (EMDB) and atomic coordinate models generated were deposited in the PDB. Below are the PDB and EMDB accession codes.
PDB entry ID 8GFT and EMDB entry ID EMD-29984 (Composite map I)
PDB entry ID 8GAE and EMDB entry ID EMD-29895 (Composite map II)
EMDB entry ID EMD-29973: Map obtained through focused classification of the PP5 catalytic domain. Consensus map I.
EMDB entry ID EMD-29976: Map obtained through focused classification of the PP5 catalytic domain. Consensus map II.
EMDB entry ID EMD-29957: Map obtained through focused classification of the PP5 TPR domain.
EMDB entry ID EMD-29949: Map obtained through focused classification of Cdc37 Middle domain.

PDB models used for model building and data analysis in manuscript and supplementary files, PDB: 8GFT, 8GAE, 5FWK, 1WAO, 5HPE, 1S95, 6Q3Q, 7KW7, 7L7I

Human research participants

Policy information about studies involving human research participants and Sex and Gender in Research.

Reporting on sex and gender	not applicable
Population characteristics	not applicable
Recruitment	not applicable
Ethics oversight	not applicable

Note that full information on the approval of the study protocol must also be provided in the manuscript.

Field-specific reporting

Please select the one below that is the best fit for your research. If you are not sure, read the appropriate sections before making your selection.

☒ Life sciences ☐ Behavioural & social sciences ☐ Ecological, evolutionary & environmental sciences

For a reference copy of the document with all sections, see nature.com/documents/nr-reporting-summary-flat.pdf

Life sciences study design

All studies must disclose on these points even when the disclosure is negative.

Sample size	Three replicates were thought necessary as internal quality checks, to account for experimental and measurement variation. Samples in which experiment got interrupted were repeated a fourth time. This number of replicates appeared sufficient after initial experiment statistical tests and were therefore deemed sufficient for the remainder of experiments. Statistical significance is reported in the manuscript.
Data exclusions	PP5 catalytic domain mutant results gave uninterpretable results, and thus provided no useful information regarding the correctness of our models. All raw western blot data is included in the supplementary. FCS allows for single molecule detection, and so large protein aggregates were excluded from analysis.
Replication	All experiments were confirmed with multiple biological replicates (n =3+).
Randomization	No randomization was performed, since there was one scientist doing experiments who had to be aware of protein being added to the tube.
Blinding	No blinding was performed in any of the biochemical experiments, since one scientist was doing experiments and had to be aware of the protein being added to the tubes. In the CryoEM work, no PP5 template was given to the initial rounds of classification in which we saw PP5 densities show up, suggesting no "einstein from noise" phenomenon has occurred.

Reporting for specific materials, systems and methods

We require information from authors about some types of materials, experimental systems and methods used in many studies. Here, indicate whether each material, system or method listed is relevant to your study. If you are not sure if a list item applies to your research, read the appropriate section before selecting a response.

Materials & experimental systems

n/a	Involved in the study
☐	☒ Antibodies
☐	☒ Eukaryotic cell lines
☒	☐ Palaeontology and archaeology
☒	☐ Animals and other organisms
☒	☐ Clinical data
☒	☐ Dual use research of concern

Methods

n/a	Involved in the study
☒	☐ ChIP-seq
☒	☐ Flow cytometry
☒	☐ MRI-based neuroimaging

Antibodies

Antibodies used	Thermo Fisher Scientific: Catalog # MA5-15176 Phospho-c-Raf (Ser338) Monoclonal Antibody (E.838.4), Catalog # MA5-33196 Phospho-c-Raf (Ser621), Recombinant Rabbit Monoclonal Antibody<br><br>Cell Signaling Technology: Phospho-CDC37 (Ser13) (D8P8F) Rabbit mAb #13248<br><br>GE Healthcare UK Limited: HRP secondary antibody Anti-Rabbit # NA9340V
Validation	Anti-Cdc37 pS13 antibody doesn't blot E. coli purified Cdc37, but blots CK2 phosphorylated Cdc37 and mammalian expressed Cdc37. pS13 antibody was also validated by Cell Signaling, for examples of antiody usage see: Nida Haider, et. al. J Clin Invest, 2021. John C Dawson, et. al. Mol Cancer Ther 2020. Ying Ao, et. al. Sci Adv 2019<br><br>Anti-CRaf pS338 antibody was validated by ThermoFisher, for examples of antibody usage see: Mitra S et al. The Journal of Biological Chemistry, 2016. Ibrahim WW et al Sci Rep 2019<br><br>Anti-CRaf p621 antibody was validated by ThermoFisher, they provide immunofluorescence proof of function, and Western Blot proof of function. More can be seen on their website: https://www.thermofisher.com/antibody/product/Phospho-c-Raf-Ser621-Antibody-Recombinant-Monoclonal/MA5-33196

Eukaryotic cell lines

Policy information about cell lines and Sex and Gender in Research

Cell line source(s)	Thermo Fisher Scientific Expi HEK293 cells from were used for complex purification. No in-cell experiments were conducted.
Authentication	The cell lines used were not authenticated, as only the purified protein was used in these studies.
Mycoplasma contamination	Cell lines were not tested for Mycoplasma contamination, as only the purified protein was used in these studies.
Commonly misidentified lines (See ICLAC register)	No commonly misidentified cell lines were used in this study.
